# Society Meetings

**Published:** 1903-11

**Authors:** 


### SOCIETY MEETINGS.

---

THE American Association of Obstetricians and Gynecologists held its sixteenth annual meeting at Chicago, September 22-24, 1903, under the presidency of Dr. L. H. Dunning, of Indianapolis. The attendance was large, the papers were of excellent quality, and the discussions of sustained interest from the beginning until the close of the sessions. The official report of the proceedings, including the papers read will be published in the *American Journal of Obstetrics* for November. The annual dinner was served at the Auditorium Annex and was an occasion of unusual interest. Among the speakers were Drs. W. A. Evans and Fernand Henrotin, Chicago; Charles L. Bonifield, Cincinnati; Norman Bridge, Los Angeles; J. H. Carstens, Detroit; W. G. Macdonald, Albany, and L. S. McMurtry, Louisville. A luncheon was tendered the association by Drs. Murphy, Ferguson, Goldspohn and Lyons, which was served at the Athletic Club on the third day of the meeting and which was a function superb in all of its appointments.

The following-named officers were elected for the ensuing year: president, Walter Blackburn Dorsett, Saint Louis; vice-presidents, Aaron B. Miller, Syracuse, and William D. Haggard, Nashville; secretary, William Warren Potter, Buffalo; treasurer, Xavier O. Werder, Pittsburg; executive committee (to fill vacancies), Rufus B. Hall, Cincinnati, and Lehman H. Dunning, Indianapolis.

---

THE Roswell Park Medical Club, at a meeting held October 12, 1903, elected officers as follows: president, Dr. F. C. Busch; vice-president, Dr. James A. Gibson; secretary and treasurer, Dr. George F. Cott.

---

THE Mississippi Valley Medical Association at its annual meeting, held at Memphis, October 7-9, 1903, elected the following-named officers: president, Hugh T. Patrick, Chicago; first vice-president, Bransford Lewis, Saint Louis; second vice-president, George

W. Cale, Springfield, Mo.; treasurer, Thos. Hunt Stucky, Louisville; secretary, Henry Enos Tuley, Louisville.

---

The Buffalo Academy of Medicine held meetings during the months of September and October, 1903, as follows:

*Section on Ophthalmology, Otology, Laryngology and Rhinology.*—Tuesday, September 29. Program: Report of cases: (1) Peculiar symptoms following radical mastoid operation; (2) Unusual case of aneurism pressing on recurrent nerve; Presentation of specimens: (1) Carcinoma of esophagus; (2) Large nasal polypus; (3) Diphtheritic tracheal casts, George F. Cott; The auditory nerve; its anatomy and physiology, James A. Gibson.

*Section on Surgery.*—Tuesday, October 6. Program: Cerebral compression due to traumatic extradural hemorrhage, Eugene A. Smith; subject unannounced, Roswell Park; Presentation of cases: Acute obstruction caused by inflammation of Meckel's diverticulum, Marshall Clinton.

*Section on Medicine.*—Tuesday, October 13. Program: The treatment of pneumonia, DeLancey Rochester. Discussion: Allen A. Jones; Hydrocephalus: an attempted classification, William C. Krauss.

*Section on Pathology.*—Tuesday, October 20. Program: Protozoa as causes of disease (yellow fever, spotted fever, smallpox, malaria and dysentery), H. G. Matzinger; The protozoon of smallpox,—illustrated by lantern slides,—G. N. Calkins.

*Section on Obstetrics.*—Tuesday, October 27. Program: Personal experience in puerperal fever, Chas. E. Congdon. Discussion opened by Herman E. Hayd.

---

The Lake Erie Medical Society will hold its next regular quarterly meeting at Dayton, N. Y., Friday, November 6, 1903. The following program has been prepared: (1) Early diagnosis and treatment of tubercular osteitis of the knee, Bernard Bartow, Buffalo; (2) The present status of treatment by *x-ray,* and ultraviolet ray, its results and restrictions, Joseph C. Clark, Olean; (3) Interference in labor, Ira W. Livermore, Gowanda; (4) Paper, L. G. Hanley, Buffalo; (5) The present status of prevention and treatment of pulmonary tuberculosis, A. D. Lake, Gowanda. The president is S. S. Bedient, Little Valley; vice-president, A. L. Borden, Gowanda; secretary and treasurer, ——

Smith, Olean; program committee, A. D. Lake, Gowanda, and C. H. Richards, Dunkirk.

---

The Medical Association of Erie County held its quarterly meeting at the University Club, Buffalo, Monday, October 5, 1903, at 8.30 p.m. The following program was observed: (1) Presentation of cases and specimens; (2) Acute hemorrhagic pancreatitis, with report of a case, Marshall Clinton. Leaders of discussion: Stephen Y. Howell, Charles G. Stockton; (3) General discussion; (4) Report of cases: (a) Perforative appendicitis in the puerperium; (b) Partial closure of the vagina due to puerperal infection, James E. King. Leaders of discussion: L. G. Hanley, L. Schroeter; (5) General discussion.

